# Implementation and Evaluation of a Training Program to Improve Patient Navigators’ Competencies: A Quasi-Experiment at a Public Tertiary Hospital in China

**DOI:** 10.3390/healthcare13040387

**Published:** 2025-02-11

**Authors:** Shuo Liu, Weiwei Tang, Qing Chang, Jueming Lei, Haitao Yue, Linjie Hou, Laura Morlock

**Affiliations:** 1 Office of Planning and Development, Peking Union Medical College Hospital (Dongdan Campus), No.1 Shuaifuyuan Wangfujing Dongcheng District, Beijing 100730, China; shuoliu@jhu.edu; 2 Human Resource Department, Peking Union Medical College Hospital (Dongdan Campus), No.1 Shuaifuyuan Wangfujing Dongcheng District, Beijing 100730, China; tweiwei220@163.com; 3 Xidan Campus Affairs Management Office, Peking Union Medical College Hospital (Xidan Campus), No.41 Damucang Hutong, Xicheng District, Beijing 100032, China; cqj9999@aliyun.com; 4 National Research Institute for Family Planning, No.12 Da Hui Si Road, Haidian District, Beijing 100081, China; blacknut2011@hotmail.com; 5 Institute of Hospital Management, Shenzhen International Graduate School, Tsinghua University, K Building, Nanshan District, Shenzhen 518055, China; yueht17@foxmail.com; 6 Institute of Health Professions Education Assessment and Reform, China Medical University, No.77, Puhe Road, Shenyang North New Area, Shenyang 110122, China; hlj8586@126.com; 7 Department of Health Policy and Management, Johns Hopkins Bloomberg School of Public Health, Johns Hopkins University, 615 N. Wolfe Street, Room W1513, Baltimore, MD 21205, USA

**Keywords:** patient navigator training, competency improvement, public tertiary hospital, healthcare service quality, Miller’s Pyramid Model

## Abstract

**Background/Objectives:** Patient navigation is vital for improving healthcare accessibility and patient experience in China’s public hospitals, where high patient demand meets limited medical resources. Patient navigators (PNs) assist patients through the complex healthcare system, but the lack of standardized training and evaluation hampers their ability to meet patient needs. This study piloted a Competencies Improvement Training Program (CITP) in a tertiary hospital to clarify PN competencies, design a feasible curriculum, assess its efficacy, and share insights with peer hospitals. **Methods:** The CITP used the Plan–Do–Check–Act (PDCA) framework and designed a curriculum with Miller’s Pyramid Model. Over 6 months, eight sessions were conducted, including theory, case studies, etc. The quasi-experimental design compared PN competencies and patient satisfaction before and after. Multiple instruments measured baseline competencies and program efficacy with a 6-month post-training follow-up. **Results:** A total of 75 PNs (75%) participated and completed all sessions. A total of 1189 patients were surveyed before training, 495 in the first month after training, and 502 in the 6-month follow-up. The CITP significantly boosted PN competency scores from 90.259 to 95.453, though it dipped to 92.721 by 6 months. Patient satisfaction with PN services improved modestly over 6 months. Challenges in applying theoretical knowledge to practical skills were noted, suggesting differentiated training based on navigator demographics. Patient satisfaction for aspects like politeness and tone was linked to patient age and education. **Conclusions:** The CITP enhanced PN core competencies and provided an evidence-based curriculum model. Future research should involve larger multi-center populations with longer-term follow-ups to validate the program’s effectiveness across diverse settings.

## 1. Introduction

Patient navigation is a patient-focused and barrier-focused intervention that aims at addressing health disparities. It achieves this by assisting individuals to eliminate barriers, overcome obstacles to care, and complete necessary healthcare services [1,2]. Pioneered by Dr. Harold Freeman, “father of patient navigation” [3], who started the first patient navigation program in the late 1980s [4], patient navigation programs have gained global traction [5]. The United States and Canada have initiated the most patient navigation programs for adults with complex needs. Such programs focus on cancer patients, especially under-served cancer patients with breast, cervical, colorectal, and prostate cancer. Patient navigation also supports other vulnerable groups, including end-of-life patients, those living with HIV, and prisoners [2,6,7,8]. These individuals often face barriers such as communication difficulties and barriers to the medical system [9]. Patient navigators (shorted as PNs), as important service providers in a healthcare delivery system, play an irreplaceable role in improving healthcare access, which is recognized as a fundamental human right [10]. In China, PNs are typically employed by hospitals or third-party outsourcing groups to provide services in their working hospitals. Their competencies are closely related to the patient experience and hospital reputation.

The World Health Organization defines competency as “the knowledge, skills, attitudes, and behaviors needed for people within an organization that are developed through education, training, and experience” in its Global Competency Framework for Regulators of Medical Products [11]. Three perspectives have emerged regarding the optimal model of the core PN tasks and competencies, each emphasizing different aspects of competencies and training (shown in Table 1). The first model focuses on certain personal qualities and the willingness to help others improve their lives [12]. The second model highlights the professional competencies and training for those navigating the screening, diagnostic, treatment, and survivorship processes [13]. The third model suggests that nonprofessional staff must be trained with one accreditation standard and documented by a recognized professional organization. The core competencies of the PNs mentioned in these three models include the ability to provide information and education to patients and others, which requires familiarity with the systems [14], as well as emotional and supportive care [6], which requires good communication skills, and the capacity to coordinate care and services.

**Figure 1:**
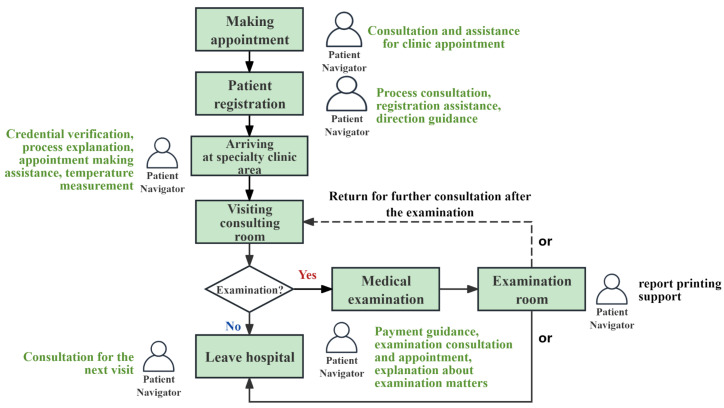
Outpatient journey and PN’s service at Xidan Campus, PUMCH.

**Figure 2:**
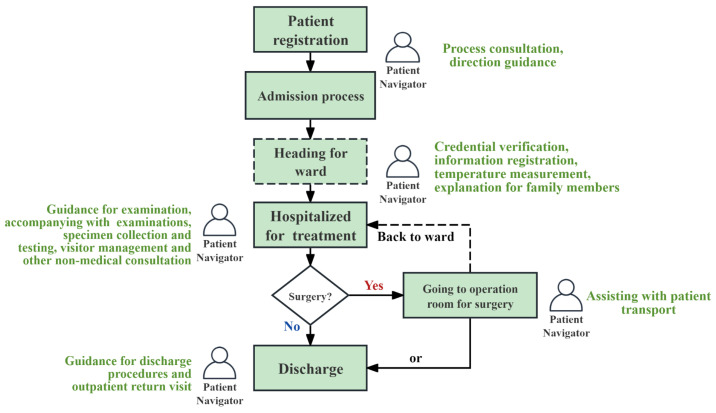
Inpatient journey and PN’s service at Xidan Campus, PUMCH.

The role of patient navigation services is gaining increasing attention in China, especially in the public healthcare sector [15,16,17]. Peking Union Medical College Hospital (PUMCH) is a Class A tertiary public hospital located in Beijing, China [18]. The Xidan Campus, one of its four branches, treated nearly 14,400 inpatients in 2021 and provided outpatient care to more than 350,000 individuals, all supported by a workforce of only approximately 500 employees [19]. The limited human resources in the hospital have led to an increasing error rate in the patient triaging process, along with more complaints about negative attitudes, unresponsive language, and lack of promptness from patient navigators. To address these issues, a human resource outsourcing company has employed 100 patient navigators to assist the hospital. Throughout the outpatient journey, PNs can assist patients in clinic appointments and registration; provide guidance in the use of self-machines; etc. See Figure 1. Throughout the inpatient journey, PNs can assist in providing guidance for examinations, accompanying in examinations, helping with discharge, etc. See Figure 2.

**Table 1:** Three models of PN core tasks and competencies.

Model	Strategy	Core Tasks and Competencies	Source
Navigator quality of care strategies and objectives	Understandable Available Accessible Affordable Appropriate Accountable	To provide education to improve knowledge, attitudes, and practices regarding cancer. To map out the location of and identify contact individuals for cancer services and to advocate for services to fill service gaps. To remove structural and cultural barriers to services, ensuring that what is available can be accessed. To ensure that individuals are enrolled in insurance and free and low-cost programs for which they are eligible so that cost is not a barrier. To establish culturally competent services and staff at facilities and programs utilized by community members. To ensure the sustainability, quality, cultural appropriateness, and responsiveness of the cancer services to and for the population(s) of focus.	[12]
Navigation through the continuum of care	Phase:outreach navigation Goal: primary prevention	Primary task: Adoption of healthy lifestyle Disease prevention	[13]
	Phase: diagnostic navigation Goal: screening early detection	Primary task: remove barriers to access to screening	
	Phase: treatment navigation Goal: antineoplastic therapy	Primary task: Education support Coordination of multi-disciplinary care Resource referrals	
	Phase: survivorship navigation Goal: supportive and tertiary care	Primary task: Wellness/nutrition Stress management Education Long-term care plans Support groups Retreats	
Core areas of practice and associated competencies for nurses working as professional cancer navigators	Building on a conceptual framework designed by [20] to define the core areas of practice and associated competencies for professional cancer navigators	Providing information and education Providing emotional and supportive care Facilitating coordination and continuity of care	[14]

**Figure 3:**
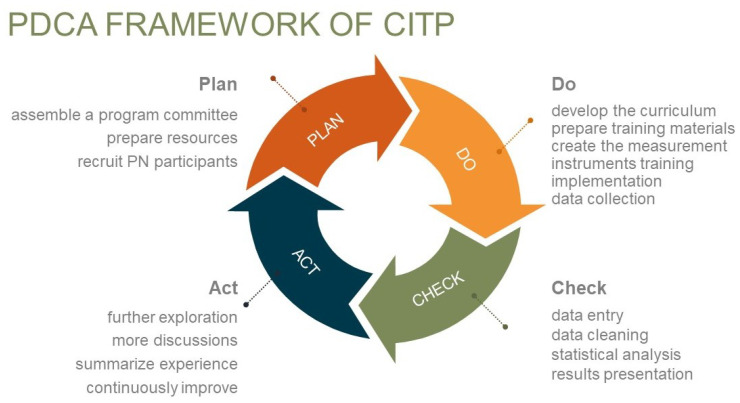
PDCA framework.

**Figure 4:**
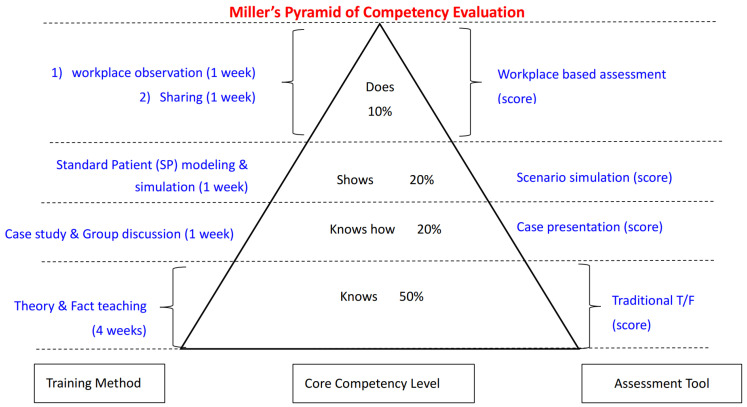
Training Design adapted from Burns and Mehay (2009), Miller’s Prism of Clinical Competency.

Although PNs receive training from their employer prior to starting their roles, there have been no hospital-initiated competency training programs or standardized evaluations. This situation results in PNs being unable to provide navigation services with more confidence, efficiency, and high quality. To clarify the core competencies of PNs in public hospitals of China, understand their current competency and potential impact factors, design a feasible training program, and evaluate its efficacy, a program called the “Competencies Improvement Training Program (CITP)” was implemented at the Xidan Campus of PUMCH in 2024. It was established as a pilot initiative for future programs in this hospital. The CITP adhered to the Plan–Do–Check–Act (PDCA) framework (see Figure 3) to keep it sustainable, and its curriculum was designed using Miller’s Pyramid Model (see Figure 4). The CITP consisted of three parts: listing the core competencies and designing the curriculum, launching a series of training modules, and evaluating the program efficacy. The results of the study show a positive impact of the CITP on improving overall PN competencies and patient satisfaction by comparing them before and after the training program.

## 2. Materials and Methods

### 2.1. Study Design

The CITP was designed as a quasi-experiment and a prospective before–after study. This study employed a Plan–Do–Check–Act (PDCA) framework and Miller’s Pyramid of Competence as the design framework for the training curriculum. The hypothesis of this study is that the CITP would improve core PN competencies and patient satisfaction related to navigation services in a certain period. The training program included 8 course sessions over 8 weeks, 1 course session per week and every session lasting 1 to 1.5 h. All sessions were face-to-face activities. All eligible PNs who gave their informed consent voluntarily participated in the program and evaluations.

PN demographic information, baseline competencies, and baseline patient satisfaction were collected prior to starting the training. Competency and learning performance measurements were made after each course session to see the change trend. The course evaluation survey was administered at the end of the last course session. Follow-up patient satisfaction and PN competencies were evaluated in the first and sixth months as post-training measures.

In this study, the variables were expressed as mean and standard deviation values. The Shapiro–Wilk test was utilized to evaluate the normality of the data distributions. Pairwise *t*-tests were carried out to compare the means between the two groups. All statistical analyses were executed using R software (version 4.2.2, R Foundation for Statistical Computing), with the help of the following packages: “tidyverse” (version 2.0.0), “ggplot2” (version 3.4.1), and “tidyr” (version 1.3.0).

### 2.2. Participants

A patient navigator could be enrolled in the program if they were employed at the Xidan Campus of PUMCH and willingly participated in all training sessions and assessments. Regarding patient participants, they were eligible to enter the study if they were 18 years or older, received services from PNs on the Xidan Campus, and had the ability to use a mobile phone application, read an online survey, and respond to it independently. In contrast, if they did not meet these criteria, they were excluded from the program. It is important to note that whether or not an individual was enrolled in the program had no bearing on their employment status (in the case of patient navigators) or their ability to receive medical treatment at this campus (in the case of patients).

Out of 100 PNs employed at the Xidan Campus of PUMCH, 75 PNs (75%) consented to take part in the program and successfully completed all sessions. In this group, 65 PNs (86.67%) were under 30 years old and 65 (86.67%) were female. The vast majority, 69 participants (92.00%), self-identified as Han Chinese. In terms of educational background, 54 participants (72.00%) held a technical school degree. Additionally, 66 participants (88.00%) were unmarried. Most of the participants (54, 72.00%) attended previous training more than three times, and 70 participants (93.33%) received previous training for more than 8 h.

A total of 1189 eligible patients voluntarily completed the satisfaction survey prior to the training program. After the training concluded, 495 eligible patients participated in the evaluation in the first month, and 502 patients took part in the follow-up evaluation 6 months after the training course was completed. The sample size was calculated using the formula $n=\left(\left(Z^{2}\right)*\left(p\right)*\left(1-p\right)\right)/E^{2}$. Subsequently, the adjusted sample size was computed as n adjusted = n/(1+((n−1)/N). For a 95% confidence interval, Z-value = 1.96, p = 0.5, E = 0.05. The value of N was calculated based on the daily average number of patient visits at the Xidan Campus.

### 2.3. Procedures

#### 2.3.1. Plan

In the planning stage, a program committee was assembled from the start to develop the curriculum, recruit PN participants, create the measurement instruments, and execute the program. The members were administrators, senior nurses, senior PNs, facilitators, and volunteers of PUMCH (see Table 2). Resources, including human resources, teaching materials, and conference rooms with remote technologies, were primarily sponsored by PUMCH.

#### 2.3.2. Do

In the “Do” stage of this program, development of the curriculum, preparation of training materials, and creation of measurement instruments were the initial steps. This phase spanned 6 months, from March to September 2024. The implementation process adhered strictly to the established curriculum. It was under the supervision of the committee to ensure compliance and quality. The training sessions, designed in accordance with the curriculum, were carried out over an 8-week period, with 1 session held each week. This structured approach allowed for a systematic and organized transfer of knowledge and skills into practice.

In the first 2 months, the curriculum (see Table 3) and training materials were designed and adjusted by committee members and external professional advisors. Miller’s Pyramid was developed specifically for assessing student competency in healthcare settings [21] and utilized as a conceptual model when designing the training curriculum. Training programs such as “Oncology Patient Navigator Training: The Fundamentals” developed by the GW Cancer Center [22], a standardized national training program supported by three institutes [2], provided curriculum design and implementation examples. Although training programs for PNs aim to improve their competencies and equip them to support patients more effectively, the tasks and competencies of PNs in real-world settings are various from one hospital to another, and even different over time. This curriculum also addressed PUMCH-defined PN competencies and patients’ needs in this hospital, and followed the Feasible, Interesting, Novel, Ethical, and Relevant (FINER) criteria.

At the foundational “knowledge” level, patient navigators were provided with comprehensive knowledge, including information about the healthcare system, the history and culture of PUMCH, patient rights, the patient journey, patient experience, communication skills, and leadership skills required as a member of an integrated healthcare team, along with an understanding of the resources available at PUMCH. Within this domain, four sessions were delivered through a combination of didactic training sessions and workshops.

The “Knows How” level entailed the application of acquired knowledge to address patient needs. This was developed through case-based learning, role-playing scenarios, and group discussions. In this domain, the CITP focused on real-world cases and equipped PNs with appropriate methods for guiding patients based on their specific needs.

The level of “Shows” was where navigators demonstrated their skills in real or simulated settings, such as mock patient interactions or supervised practical training. One session consisting of real cases and opportunities for all PN participants to show their solutions was provided.

Finally, the ‘Does’ level pertained to the actual performance of navigators in their day-to-day work, which was evaluated through continuous supervision and feedback. All participants applied what they had learned and practiced in the preceding sessions to their real work. To gather feedback from PN participants regarding the program and encourage them to share their thoughts during the training, an additional sharing session was incorporated into this domain.

The success of training programs built on Miller’s Pyramid is affected by the quality of implementation, which encompasses the utilization of evidence-based information, case studies, and continuous evaluation and feedback [21,23]. Various learning modalities were used, such as video tutorials, traditional lectures, in-depth case studies, engaging role-plays, and realistic simulations. This diverse set of methods was used to enhance the learning experience and ensure comprehensive knowledge acquisition and skill development [24,25,26], as well as workplace practice. Participants were actively encouraged to engage with the program through various means, such as participating in Q and A sessions, engaging in group discussions, delivering class presentations, and attending debriefing sessions. These activities enabled them to interact with instructors and share ideas with one another, thereby enhancing their understanding and collaborative learning experience [27,28,29].

#### 2.3.3. Check

All assessments were implemented following the study design. Each PN participant was given a study ID, which was used in the data collection process instead of using any identifiable information. Researchers who interacted with participants during data collection did not know their identity or study ID. This was achieved through careful data coding and strict separation of information channels. The instruments used for data collection were based on those developed by relevant previous programs that had been assessed for reliability and validity; there were only minor adjustments to the instruments for this study. The results were then presented and discussed at a CITP Committee seminar, facilitating the exchange of ideas and insights among the participants. Follow-up evaluations were performed 6 months after training to assess whether improvements in competencies and patient satisfaction were sustained. Data collection was carried out in a controlled environment. Two seminars were held in the recruitment process for the 6-month follow-up, and trained research assistants were present to ensure that the participants understood the instructions clearly. For the online patient satisfaction survey, detailed instructions were provided at the beginning, and participants had also options to ask for clarification if needed. All data were entered by two research assistants in a double-cross-check process.

#### 2.3.4. ACT

The evaluation of the CITP offers an evidence base for the enhancement of future programs. We will draw on the experience and lessons learned from this pilot program to refine future programs, thus continuously improving the competency and quality of PN service.

### 2.4. Instruments

**Figure A1:**
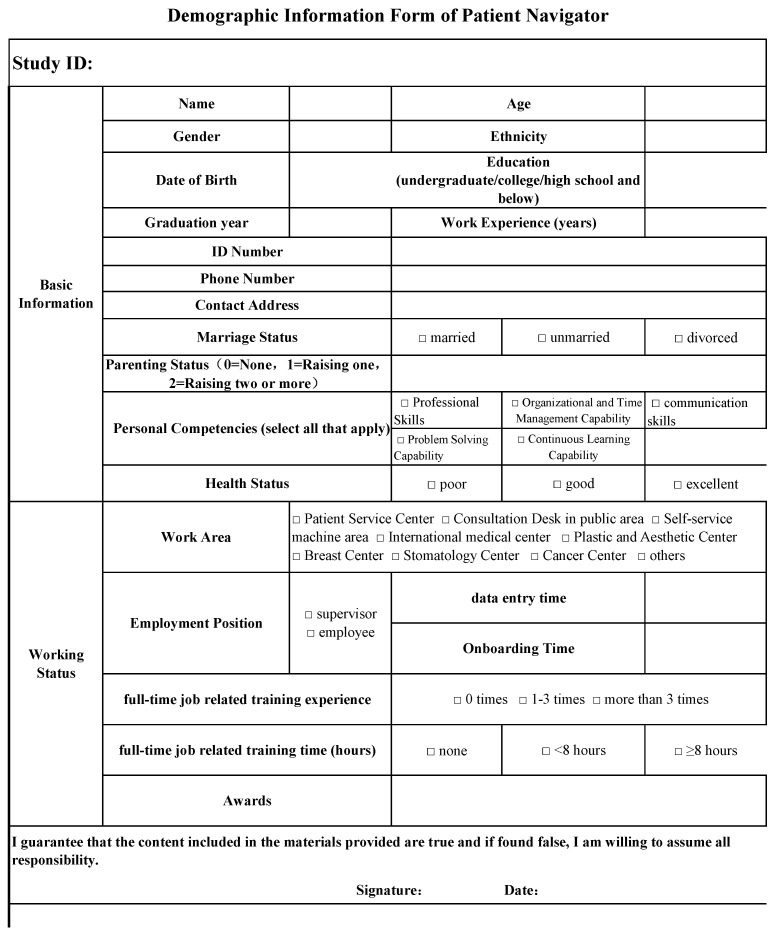
Demographic Information Form of Patient Navigators.

The Demographic Information Form for Patient Navigators (Figure A1) was designed by our team internally according to the specific requirements of the program. This custom-designed form ensures that it captures all the relevant demographic data necessary for the evaluation and understanding of the characteristics of patient navigators participating in the program.

**Figure A2:**
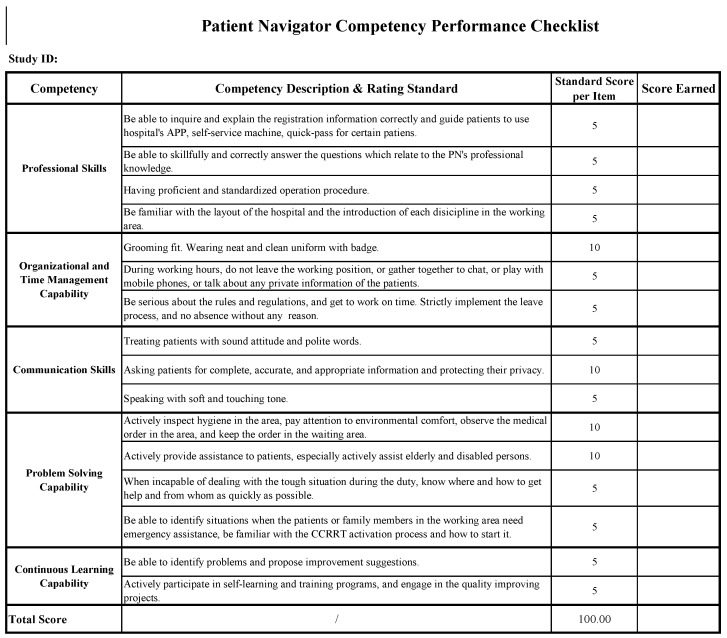
Patient Navigator Competency Performance Checklist. *Note:* In weeks 1–6 and 8, this checklist was completed by PNs. In week 7, this checklist was completed by instructors.

The Competency Rating Form (Figure A2) was designed by the committee members, drawing on other PN training programs, such as the National Cancer Institute Patient Navigation Research Program [30], the National Patient Navigator Training Program [2], the list of patient navigator competencies of Western Governors University [31], and Professional Patient Navigator Competencies by the American Cancer Society [32]. This approach ensures that the form benefits from established best practices and relevant precedents, helping to make the evaluation more comprehensive and reliable.

**Figure A3:**
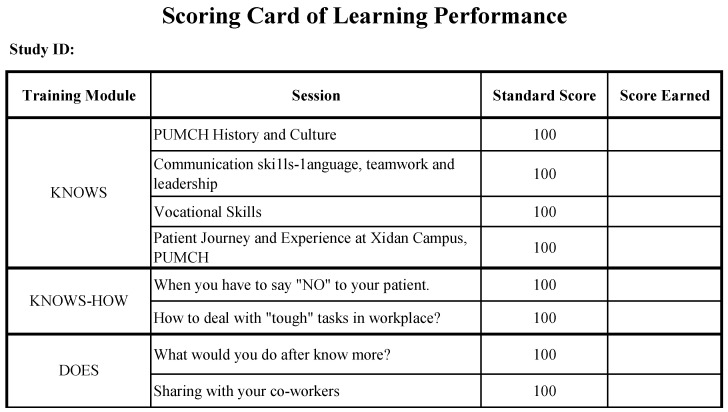
Scoring Card of Learning Performance. *Note:* This scoring card was completed by instructors.

The Scoring Card of Learning Performance (Figure A3) was designed by all trainers, taking into account the specific requirements of each training session. This ensures that the card accurately reflects the performance criteria relevant to the learning outcomes of the training sessions, enabling a comprehensive and accurate assessment of participants’ progress.

**Figure A4:**
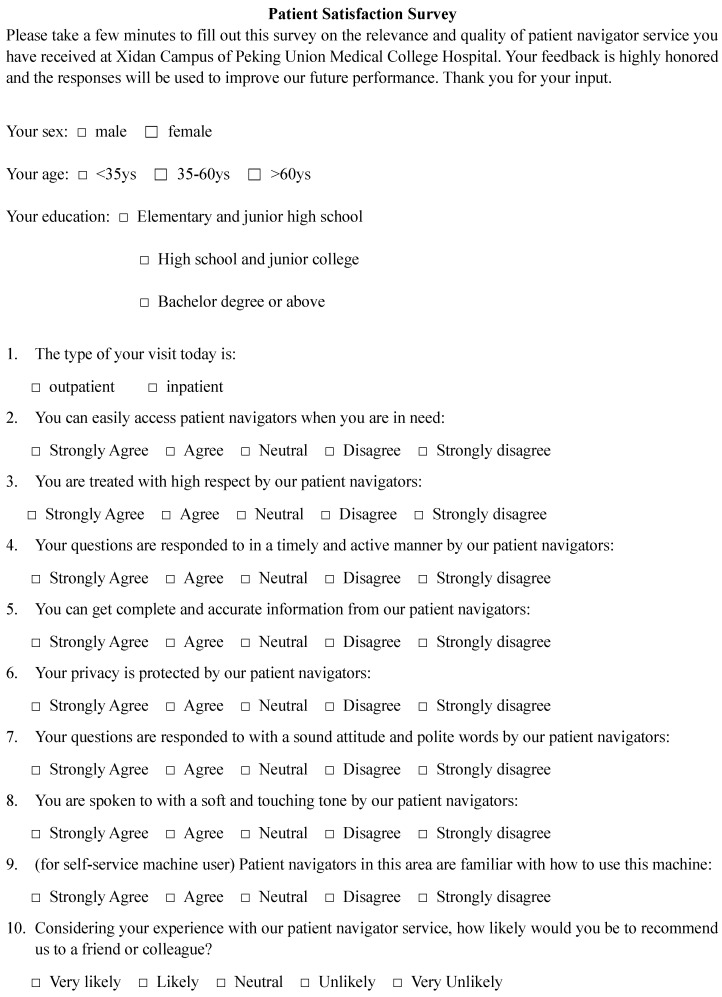
Patient Satisfaction Survey.

The Patient Satisfaction Survey (Figure A4) was designed based on previous research [15] and the official requirements of the Chinese National Health Commission [17] with minor improvements suitable for PUMCH.

**Figure A5:**
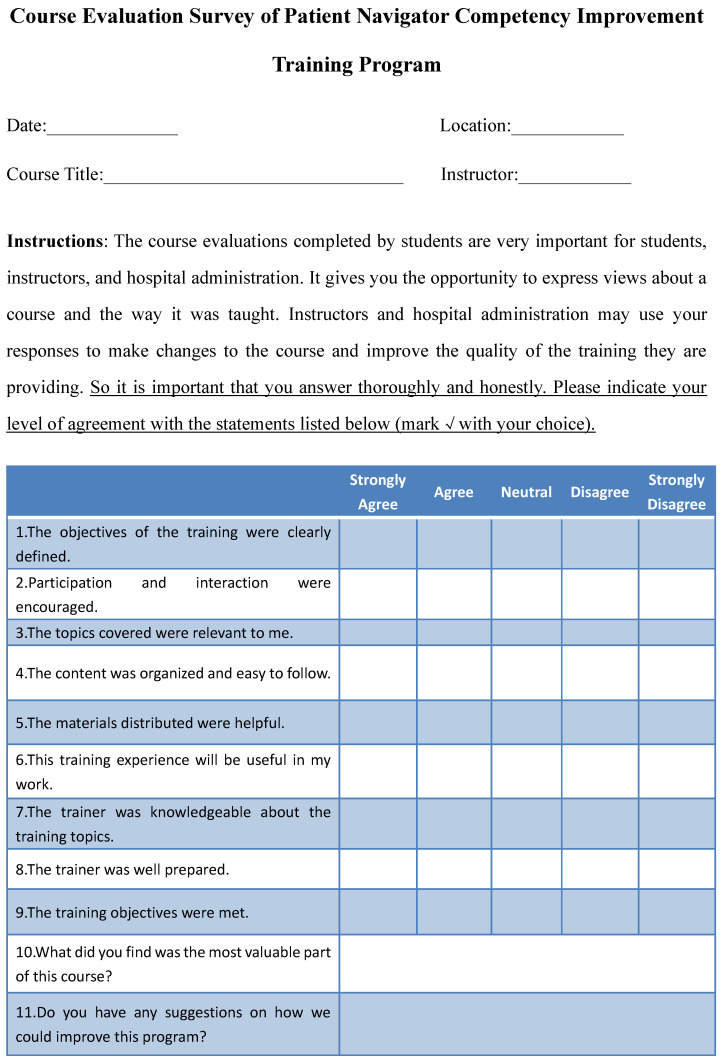
Course Evaluation Survey.

The Course Evaluation Survey (Figure A5) was designed using a 5-point Likert scale that ranges from “strongly agree” to “strongly disagree”. This design was influenced by and referenced other university student learning assessments, such as those used at the renowned Johns Hopkins University [33]. It aims to provide a comprehensive and standardized method for evaluating each course session, drawing on established practices from other educational institutions to ensure reliability and validity in the assessment process.

## 3. Results

**Figure 5:**
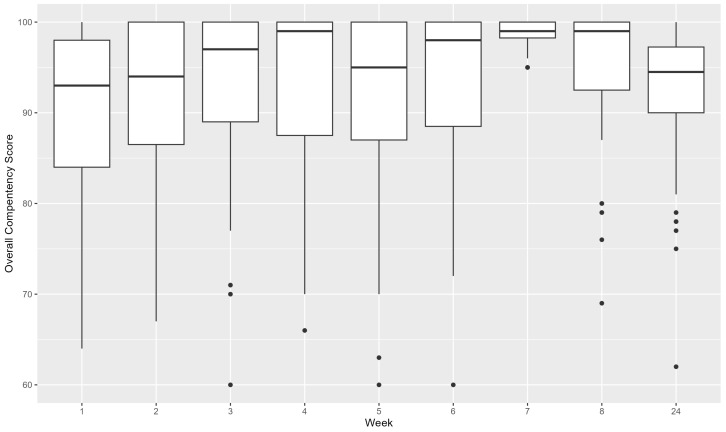
Overall competency score by week.

Figure 5 illustrates the progression of overall competency scores throughout the comprehensive training program. During the first 4 weeks, which emphasized theoretical learning, a significant upward trend was observed in the scores. However, in the fifth week, during the application phase, a decrease in PN competency scores was observed. This suggests that advancements in theoretical knowledge may not directly translate into improved application skills. Following the case study in the fifth week and the drilling performance in the sixth week, the practice-oriented segment in the seventh week produced remarkable results, with the PNs achieving an average competency score of 98.92 (95% CI 98.62–99.23), indicating exceptional performance. However, a decrease in competency scores was observed 6 months after the end of the training program (mean 92.72, 95% CI 90.99–94.45).

Table 4 presents the pre-training, post-training, and 6-month follow-up competency scores of PNs in various demographic subgroups. Moreover, paired sample t-tests were performed to assess the differences between pre-training and post-training scores, and also between pre-training and 6-month follow-up scores. The normality assumptions for the competency scores at pre-training, post-training, and 6-month follow-up were evaluated by means of the Shapiro–Wilk test. The results showed that *p* > 0.05, which verified that the data followed a normal distribution (as can be seen in Figure A13 and Figure A14).

The overall competency score of PNs increased significantly from 90.259 to 95.453 right after the training (*p* < 0.001). Nevertheless, after 6 months, there was no evidence of a lasting improvement. The mean difference between the pre-training and 6-month follow-up scores was 1.979, with a *p*-value of 0.205, suggesting that the change was not statistically significant. These results demonstrate that although all subgroups showed immediate post-training enhancements in competency scores, the majority of these improvements were not maintained over time. The interaction between sex and highest degree is presented in Table 4; however, no significant interaction effects were observed.

To ensure the validity of our findings, we examined baseline characteristics in key demographic subgroups, including gender, age, and level of education. Older PNs (>=30 years) demonstrated greater improvements compared with their younger counterparts. Married participants exhibited significantly higher post-training gains. PNs with technical school education achieved substantial progress. The results of these comparisons are summarized in Table A2, and individual figures are included in the Appendix A.

Table 5 displays patient satisfaction with the quality of PN services before and after training. Compared with the pre-training scores, the post-training scores exhibited a slight increase in the percentage of satisfaction for male patients, with a decrease for female patients. The satisfaction distribution by age also shifted, with an increase for younger patients and a decrease for those over 60 years of age. The results indicate a rise in the proportion of satisfaction for patients with a middle/junior high school education. The overall satisfaction scores remained high and were even higher (exceeded 99%) after 6 months of training completion. Fluctuations were observed, especially in Q6 (politeness) and Q7 (gentle tone), during the first month after training. However, in the subsequent 5-month period, these metrics showed an upward trend. Further details and discussion can be found in the Section 4.

Table 6 shows the results of paired sample t-tests for the pre- and post-training test scores of PNs in the first four sessions of the “Knows” module. The results indicate that the test scores of PNs significantly increased after the training. For week 5 to week 8, only post-training test scores were collected according to the course design. The performance in the “Knows-How” and “Sharing” modules indicates moderate score levels, while the “Does” and “Practicing” modules show a high level.

The results of the PN course evaluation survey indicate that participant feedback on the comprehensive training program was largely positive. In week 1, 98.67% (74/75) of the participants reported satisfaction with the training. This was followed by 97.26% (71/73) in week 2 and 97.34% (73/75) in week 3. Satisfaction peaked at 100.00% (70/70) in week 4. In week 5, 95.77% (68/71) of the participants were satisfied, 97.22% (70/72) in week 6, 96.67% (58/60) in week 7, and 98.46% (64/65) in week 8. Furthermore, some PNs reported that the training courses felt redundant and expressed a preference for more video learning. Detailed participant feedback from week 1 to week 8 can be found in Table A1.

## 4. Discussion

Overall, the CITP has significantly enhanced the competencies of patient navigators, the quality of patient navigation services, and patient satisfaction. However, upon closer inspection of these results, certain findings have piqued the authors’ interest and merit further discussion.

First, the gap between knowing and practicing exists and affects the improvement of competencies, and practice-based teaching strategies are needed. The competency score did not increase consistently (with a slope greater than 1), but showed a noticeable decline at week 5 and a rebound at week 6. One possible reason is that during the “Knows” module, where the emphasis was on theoretical knowledge, the trainees “believed” they had acquired the relevant competencies. However, in the “Knows-How” module, they became aware that they still had weaknesses and deficiencies in their competencies. This speculation was corroborated by the feedback from the participants during the final “sharing” session. Some participants voiced their concerns regarding the transition from knowledge-based learning to real-world practice. Nevertheless, through case studies, role-playing-based simulations, and workplace practice, their competency scores significantly improved. This indicates the importance of incorporating more practice-based didactic strategies into future training programs to enhance learning outcomes.

**Figure A11:**
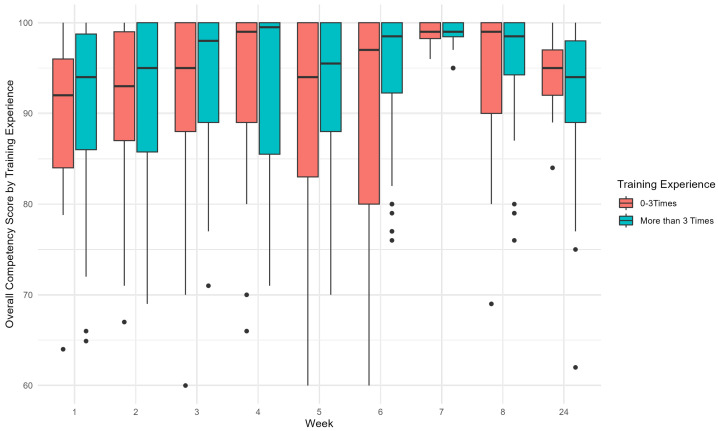
Overall competency score by training experience.

**Figure A13:**
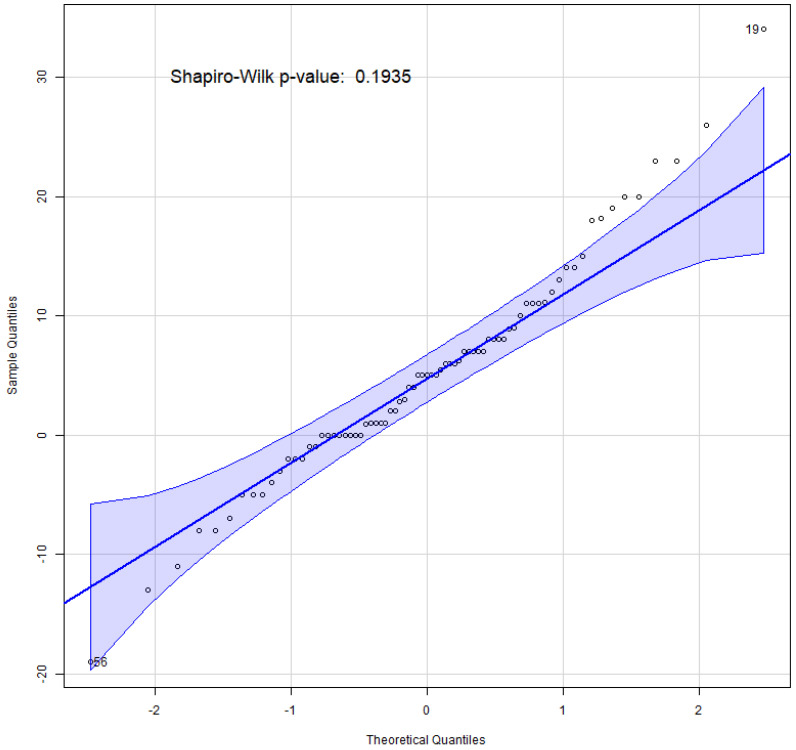
Normality check of the differences between post- and pre-training.

**Figure A14:**
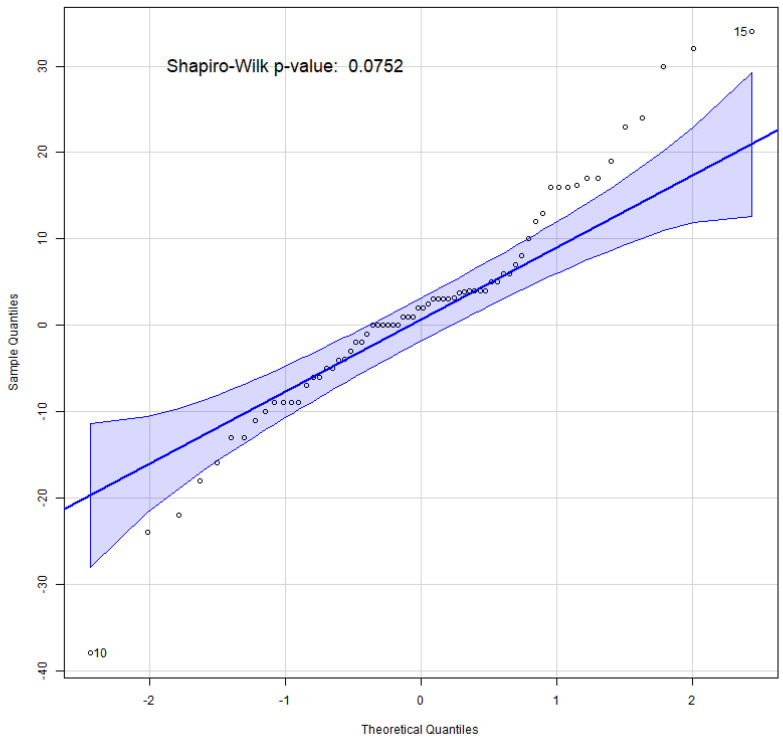
Normality check of the differences between post- (6 months) and pre-training.

Second, factors such as age, gender, educational attainment, previous training experience, and training duration can exert an impact on training performance. Older PNs have more life experience, which may allow them to understand and apply training content better in some knowledge-based trainings [34]. Female trainees showed a greater difference in competency scores before and after training and more engagement in role-playing, group discussions, and team presentations. In training such as nursing and customer service, women may show more patience and care, and be better at communicating with others [35]. This was also seen in other patient navigation programs [36]. PNs with a minimum of 8 h of prior training experience exhibited notably higher competency scores in both theory and skill-teaching aspects when contrasted with those having fewer training hours. Nevertheless, during the practical sessions, their scores were comparatively lower. Interestingly, the inflection point emerged between the fourth and fifth weeks, marking a significant shift in performance trends (see Figure A11). Trainees with relevant experience have already mastered some basic skills and knowledge, which help them to quickly engage in training [37].

Third, the differences in age and education of the patients suggest varying navigation requirements and affect patient satisfaction with PN services. Although most of the patients were satisfied with navigation service, and satisfaction generally remained high 1 month and 6 months after the completion of the training program, fluctuations in satisfaction were observed in questions related to PN politeness (Q6) and the use of gentle tones (Q7). Younger patients and those with lower levels of education tended to be more satisfied with a quick response and a polite and gentle tone of voice when receiving services. This pattern is consistent with other research findings [38,39].

Finally, a follow-up evaluation of patient navigator (PN) competency and patient satisfaction 6 months after the completion of training offers positive evidence of the sustainable efficacy of the PN training program. PNs are likely to have more chances to practice and hone the skills they acquired, which leads to continuously higher patient satisfaction. However, the difference in competency scores between the pre-training and the 6-month post-training periods shows no statistical significance. This implies that the training effectiveness based on self-assessment diminishes over time. This finding serves as a reminder that at least one additional training session is required within 6 months after the initial training to stabilize the training effectiveness. Although some PN programs, such as the Susan G. Komen’s Patient Navigation Training Program [40], have taken into account the frequency and duration of training, their effect on maintaining training outcomes needs to be further studied.

### 4.1. Implications

There are many implications of these study findings for our future research and program development as well as for peer hospitals in China.

First, when designing the program, a controlled study, whether conducted across different campuses or at multiple sites, would be a powerful option for identifying more influential factors and correlations between PN competencies and service quality outcomes. One example of such a study is the nine-site National Cancer Institute Patient Navigation Research Program (PNRP) [30]. Ongoing training in order to sustain high levels of competency is highly suggested. Future research could more effectively address issues regarding training frequency and duration.

When designing the curriculum, it is advisable to take into account the variation in requirements of PNs and patients. Trainees with higher education and previous training levels have been observed to experience substantial improvements in their competencies after training. These trainees tend to prefer more challenging programs, as indicated by course feedback. Although the majority of PNs had a positive response to the program, some provided feedback highlighting certain areas for improvement; for example, some felt that the sessions were overly long, that more video content should be incorporated, and that the program was too challenging enough.

For future programs, several modifications could be implemented. First, the duration of each session could be shortened to address the concern of excessive length. Second, integrating more video-based learning could enhance the learning experience. Additionally, the program’s difficulty level should be adjusted appropriately. A multi-level design might be put into practice to more effectively target PNs with different competency levels. Modules that are closely related to PN responsibilities and patient expectations, such as case studies, simulations, and practical exercises, should be given more attention.

When processing the competency evaluation, the “self-assessment by PNs” demonstrated a high degree of credibility. The trainees were able to honestly and realistically evaluate their own abilities, thus showing a high level of self-awareness, particularly when the training was in line with their requirements and expectations.

When practicing patient navigation activities, hospital leadership should further segment the patient population and pay attention to age distribution when creating “patient profiles,” and offer varying navigation services. PN supervisors should tailor training to enable PNs to quickly identify the personalized needs of patients. For instance, cancer patients face numerous barriers throughout the healthcare system, which means they especially require a patient navigation service. Individual and continuous assistance can significantly ease patients’ medical journey, making it more manageable for them [41]. Programs specifically aimed at navigators serving cancer patients are practical and promising.

### 4.2. Limitations

A single-site intervention and the absence of a control group may result in limitations regarding the assessment of the program efficacy. However, the inclusion of a control group of PNs who receive no training could potentially harm the interests of those patients who are served by navigators who had no opportunity to participate in the program. In addition, the limited sample size may not be representative of the overall patient navigator training landscape and could thereby affect the generalization of the findings. Although, in this study, all eligible PNs were strongly encouraged to participate in the program to maximize the sample size and program coverage, future studies should include multiple sites, which could make results more generalizable.

Due to the limited measurement instruments and methods, correlation analyses between each lesson and each core competency were not conducted in this study. This limitation significantly hampered our ability to make more accurate and targeted improvements to training strategies. There is a need to develop more sensitive instruments based on the existing research of training programs implemented in healthcare settings and other fields.

Potential biases, such as selection bias due to voluntary participation, which may lead to self-selection by more motivated PNs, and social desirability bias, where participants may report higher satisfaction in course evaluations due to perceived social and professional obligations, could affect the analysis. It is essential to acknowledge and address these potential biases in the participant selection process and analysis of survey responses. We made efforts to mitigate these threats through anonymized surveys and by encouraging all eligible PNs to participate via group-by-group and one-by-one communication during the recruiting process.

## 5. Conclusions

Chinese patients are more inclined to seek medical treatment from large public hospitals by their own choice rather than being referred by primary care physicians. The contradiction between the immense demand for medical resources in public hospitals and the limited medical supply is becoming increasingly intense. Public hospitals must continuously meet the basic medical needs of patients, enhance patient satisfaction, and ensure the physical and mental health of medical staff. All of these pose significant challenges to hospital management. Providing navigation services to assist patients through the complex medical process has emerged as an important strategy. Comparing the best practices globally, the role of navigators in China’s healthcare system is relatively new, and research on PN competencies in public hospitals is in its early stages.

The CITP, as an exploratory small-scale training project, took advantage of international experience while starting from the context of China. It was initiated and implemented by one of the most typical public hospitals. This project defined core PN competencies, designed a training model and a feasible curriculum, and developed a series of measurement instruments for the evaluation of learning outcomes. This program has been demonstrated to have a positive impact on enhancing the core competencies of patient navigators and improving the navigation services they provide to patients. Its development process and practice provide experience and implications for future programs, while more research involving a larger-scale population across multiple centers and featuring a longer-term follow-up evaluation is needed to confirm the program’s effectiveness in diverse environments. More attention should be paid to addressing variations in patient needs in daily practice and in training in patient navigator competencies.

## Figures and Tables

**Table 2:** Roles and responsibilities of CITP Committee.

Committee Member	Number	Source	Role and Responsibility
Leadership Team	4	PUMCH Xidan Campus	Lead the program, organize patient navigators to participate in the program, and coordinate internal and external resources.
Instructors	12	PUMCH, Senior PN	Teach, conduct tests, and recommend improvements.
Facilitators	2	PUMCH, Internships	Assist in organizing training activities, collecting surveys, and inputting data.
Hospital Staff Volunteers	3	PUMCH	Volunteer in organizing and collecting surveys.

**Table 3:** CITP curriculum.

Domain	Topic	Duration	Teaching Method
KNOWS	PUMCH culture and history	1 week	theory and fact teaching
	Communication skills-language, teamwork, and leadership	1 week	theory and fact teaching
	Vocational skills	1 week	theory and fact teaching
	Patient journey and experience at Xidan Campus, PUMCH	1 week	theory and fact teaching
KNOWS-HOW	When you have to say “NO” to your patient	1 week	case study, role-playing, and group discussion
	How to deal with “tough” tasks in workplace	1 week	real-world case simulation
DOES	What would you do after knowing more	1 week	workplace observation
	Sharing with your co-workers	1 week	Sharing

**Table 4:** Navigator pre- and post-training paired sample *t*-tests.

	Pre-Training				Post-Training				After 6 Months				Diff. Between Post- and Pre-Training					Diff. Between After- and Pre-Training			
Characters	Mean	SD	N		Mean	SD	N		Mean	SD	N		Mean	Sd	N	*p*-Value		Mean	Sd	N	*p*-Value
**Overall**	90.259	9.524	75		95.453	6.725	75		92.721	7.264	68		5.195	9.128	75	0.000		1.979	12.748	68	0.205
**By Age**																					
*>=30 Years*	87.980	6.529	10		98.100	3.446	10		92.333	6.614	9		10.120	5.993	10	0.000		4.800	8.100	9	0.113
*<30 Years*	90.609	9.897	65		95.046	7.025	65		92.780	7.409	59		4.437	9.322	65	0.000		1.549	13.314	59	0.375
**By Gender**																					
*Female*	90.640	9.128	65		96.062	5.900	65		92.633	7.234	60		5.422	8.874	65	0.000		1.773	12.453	60	0.274
*Male*	87.780	12.061	10		91.500	10.212	10		93.375	7.963	8		3.720	11.055	10	0.315		3.525	15.662	8	0.545
**By Ethnicity**																					
*Han*	90.281	9.420	69		95.261	6.930	69		93.290	6.176	62		4.980	8.961	69	0.000		2.477	11.713	62	0.101
*Other*	90.000	11.645	6		97.667	3.141	6		86.833	14.006	6		7.667	11.535	6	0.164		−3.167	21.656	6	0.735
**By Highest Degree**																					
*Junior College*	88.977	8.242	13		93.769	7.452	13		95.667	4.163	12		4.792	10.542	13	0.127		6.108	6.862	12	0.010
*Technical School or Below*	90.663	10.108	56		95.768	6.758	56		91.700	7.931	50		5.105	9.014	56	0.000		0.498	13.935	50	0.802
*Undergraduate*	89.267	6.952	6		96.167	4.956	6		95.333	3.615	6		6.900	8.283	6	0.097		6.067	9.390	6	0.174
**By Marital Status**																					
*Married*	87.533	6.761	9		98.111	3.655	9		92.333	6.614	9		10.578	6.169	9	0.001		4.800	8.100	9	0.113
*Unmarried*	90.630	9.822	66		95.091	6.981	66		92.780	7.409	59		4.461	9.252	66	0.000		1.549	13.314	59	0.375
**By Training Experience**																					
*0–3 Times*	88.986	8.917	21		94.190	8.183	21		94.579	4.363	19		5.205	7.942	21	0.007		4.805	9.495	19	0.041
*More Than 3 Times*	90.754	9.786	54		95.944	6.083	54		92.000	8.039	49		5.191	9.619	54	0.000		0.884	13.735	49	0.654
**By Training Length**																					
*Less Than 8 h*	91.800	6.573	5		95.800	5.848	5		95.250	4.573	4		4.000	11.424	5	0.477		0.750	6.500	4	0.832
*More Than 8 h*	90.149	9.726	70		95.429	6.820	70		92.563	7.396	64		5.280	9.037	70	0.000		2.056	13.066	64	0.213
**By Gender, Highest Degree Interaction**																					
Female*Junior College	89.700	8.716	11		94.909	6.789	11		95.818	4.332	11		5.209	11.316	11	0.158		6.118	7.197	11	0.018
Male*Technical School or Below	89.857	13.934	7		91.857	11.276	7		93.000	9.381	6		2.000	11.733	7	0.668		1.000	17.390	6	0.893
Female*Technical School or Below	90.778	9.628	49		96.327	5.822	49		91.523	7.822	44		5.549	8.619	49	0.000		0.430	13.641	44	0.836
Female*Undergraduate	91.360	5.249	5		96.000	5.523	5		95.400	4.037	5		4.640	6.888	5	0.206		4.040	8.911	5	0.368
Male*Junior College	85.000	4.243	2		87.500	10.607	2		94.000		1		2.500	6.364	2	0.677		6.000		1	
Male*Undergraduate	78.800		1		97.000		1		95.000		1		18.200		1			16.200		1	

**Table 5:** Patients’ satisfaction evaluation of PNs.

	Before Training		After Training		After Training 6 Months	
**Total number (n%)**	n = 1189		n = 495		n = 502	
**Gender**						
Male	385	32.38%	186	37.58%	183	36.45%
Female	804	67.62%	309	62.42%	319	63.55%
**Age**						
18–34	546	45.92%	247	49.90%	258	51.39%
35–60	518	43.57%	210	42.42%	190	37.85%
Above 60	125	10.51%	38	7.68%	54	10.76%
**Education level**						
Junior and middle school	112	9.42%	62	12.53%	68	13.55%
High school or equal	562	47.27%	211	42.63%	241	48.01%
Above bachelor degree	515	43.31%	222	44.85%	193	38.45%
**Question satisfaction(agree and strong agree)**						
Q1.You can easily access patient navigators when you are in need	1176	98.91%	493	99.60%	499	99.40%
Q2.You are treated with high respect by our patient navigators	1178	99.07%	491	99.19%	497	99.00%
Q3.Your questions are responded to in a timely and active manner by our patient navigators	1176	98.91%	493	99.60%	497	99.00%
Q4.You can get complete and accurate information from our patient navigators	1173	98.65%	491	99.19%	498	99.20%
Q5.Your privacy is protected by our patient navigators	1181	99.33%	492	99.39%	498	99.20%
Q6.Your questions are responded to with a sound attitude and polite words by our patient navigators	1180	99.24%	490	98.99%	498	99.20%
Q7.You are spoken to with a soft and touching tone by our patient navigators	1179	99.16%	490	98.99%	497	99.00%
Q8.Patient navigators in this area are familiar with how to use this machine	1178	99.07%	493	99.60%	499	99.40%
Q9.Considering your experience with our patient navigator service, how likely would you be to recommend us to a friend or colleague	1170	98.40%	489	98.79%	498	99.20%

**Table 6:** Test score results for course content.

Course Module	Week	Number of Participants	Pre-Train Average	Post-Train Average	*p*-Value
Knows (Theory)	1	67	61.11	77.77	<0.05
	2	73	16.67	83.35	<0.05
	3	72	66.68	100	<0.05
	4	72	58.8	100	<0.05
Knows-How (Case Study)	5	67	78.8		/
Does (Demonstration)	6	72	93.9		/
Practicing	7	71	98.92		/
Sharing	8	75	86.5		/

**Figure A6:**
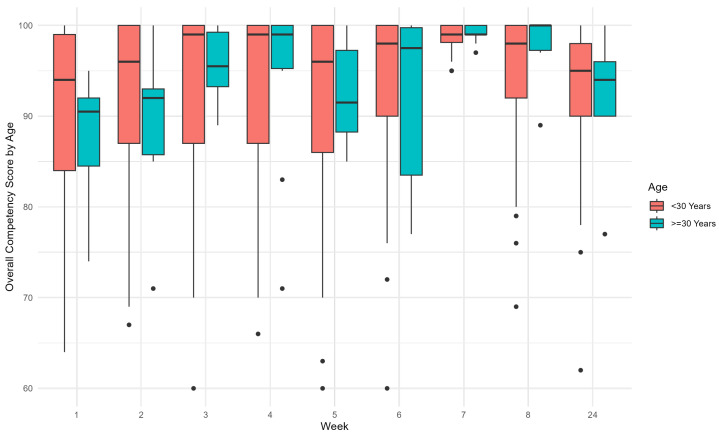
Overall competency score by age.

**Figure A7:**
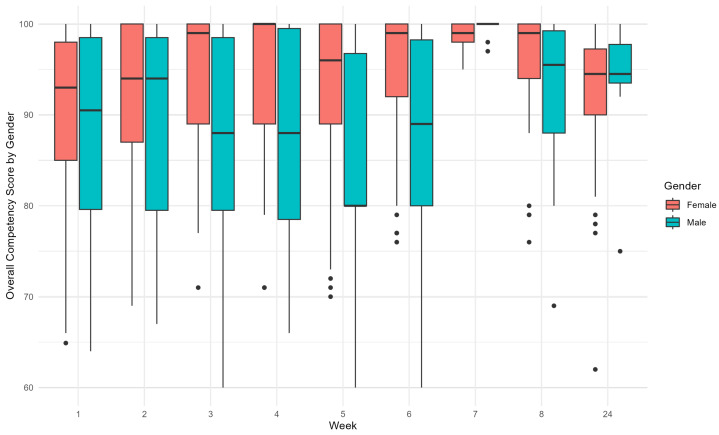
Overall competency score by gender.

**Figure A8:**
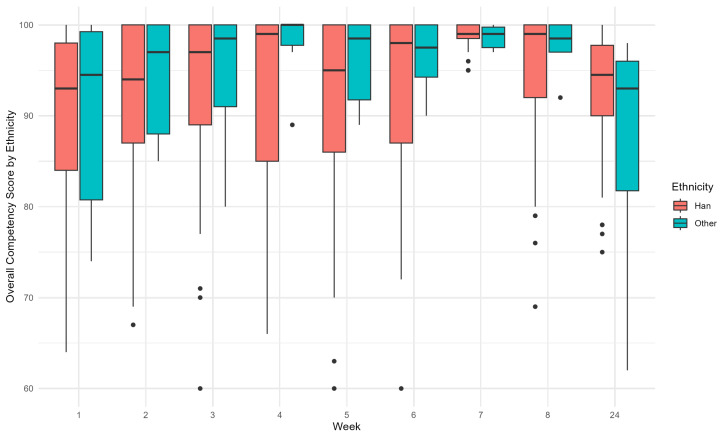
Overall competency score by ethnicity.

**Figure A9:**
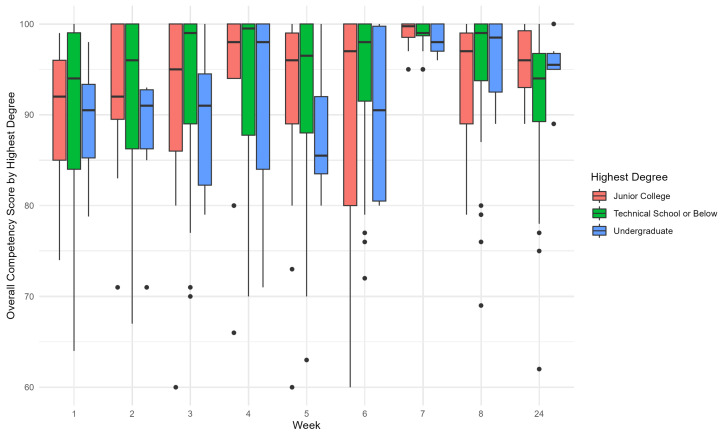
Overall competency score by highest degree.

**Figure A10:**
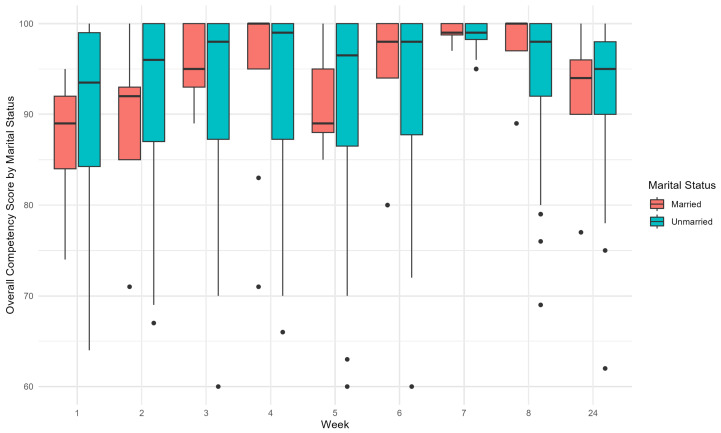
Overall competency score by marital status.

**Figure A12:**
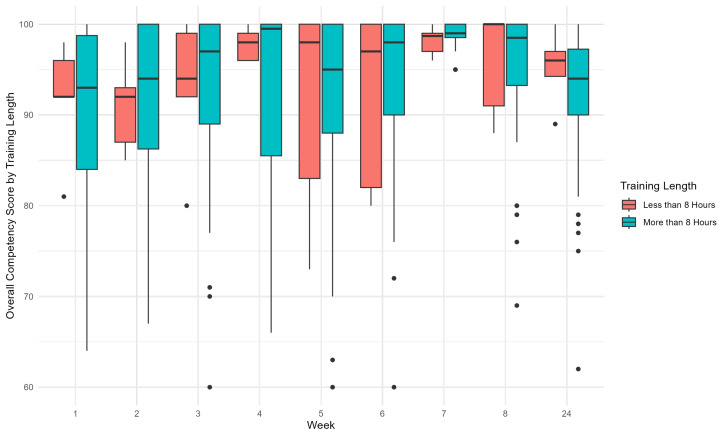
Overall competency score by training length.

## Data Availability

The raw data supporting the conclusions of this article will be made available by the authors on request.

## Abbreviations

The following abbreviations are used in this manuscript:

PN	patient navigator
PUMCH	Peking Union Medical College Hospital
CITP	Competencies Improvement Training Program

## Appendix A

**Table A1:** PNs’ feedback regarding week 1 to week 8 course sessions.

**Question** **(Week 1)**	**Strongly** **Agree**	**Agree**	**Neutral**	**Disagree**	**Strongly** **Disagree**		**Question** **(Week 2)**	**Strongly** **Agree**	**Agree**	**Neutral**	**Disagree**	**Strongly** **Disagree**
Q1	57	18	0	0	0		Q1	58	13	2	0	0
Q2	54	21	0	0	0		Q2	57	13	3	0	0
Q3	56	15	4	0	0		Q3	56	16	1	0	0
Q4	56	19	0	0	0		Q4	60	13	0	0	0
Q5	51	20	4	0	0		Q5	57	16	0	0	0
Q6	53	27	4	1	0		Q6	57	14	2	0	0
Q7	59	16	0	0	0		Q7	58	13	2	0	0
Q8	60	15	0	0	0		Q8	61	11	1	0	0
Q9	55	19	1	0	0		Q9	60	11	2	0	0
Total number					75		Total number					73
												
**Question** **(Week 3)**	**Strongly** **Agree**	**Agree**	**Neutral**	**Disagree**	**Strongly** **Disagree**		**Question** **(Week 4)**	**Strongly** **Agree**	**Agree**	**Neutral**	**Disagree**	**Strongly** **Disagree**
Q1	61	12	2	0	0		Q1	57	12	1	0	0
Q2	61	12	2	0	0		Q2	56	14	0	0	0
Q3	61	14	0	0	0		Q3	58	12	0	0	0
Q4	61	13	1	0	0		Q4	56	14	0	0	0
Q5	62	12	1	0	0		Q5	56	14	1	0	0
Q6	61	13	1	0	0		Q6	55	15	0	0	0
Q7	65	9	1	0	0		Q7	56	13	1	0	0
Q8	62	13	0	0	0		Q8	58	12	0	0	0
Q9	62	11	2	0	0		Q9	56	14	0	0	0
Total number					75		Total number					70
												
**Question** **(Week 5)**	**Strongly** **Agree**	**Agree**	**Neutral**	**Disagree**	**Strongly** **Disagree**		**Question** **(Week 6)**	**Strongly** **Agree**	**Agree**	**Neutral**	**Disagree**	**Strongly** **Disagree**
Q1	58	10	3	0	0		Q1	55	17	0	0	0
Q2	56	12	2	1	0		Q2	56	14	2	0	0
Q3	54	16	1	0	0		Q3	55	14	3	0	0
Q4	56	14	1	0	0		Q4	52	18	2	0	0
Q5	57	12	2	0	0		Q5	54	16	2	0	0
Q6	56	14	1	0	0		Q6	54	15	2	1	0
Q7	58	11	2	0	0		Q7	53	17	2	0	0
Q8	57	13	1	0	0		Q8	54	15	3	0	0
Q9	53	15	2	1	0		Q9	51	19	2	0	0
Total number					71		Total number					72
												
**Question** **(Week 7)**	**Strongly** **Agree**	**Agree**	**Neutral**	**Disagree**	**Strongly** **Disagree**		**Question** **(Week 8)**	**Strongly** **Agree**	**Agree**	**Neutral**	**Disagree**	**Strongly** **Disagree**
Q1	48	10	2	0	0		Q1	51	14	0	0	0
Q2	48	11	1	0	0		Q2	51	14	0	0	0
Q3	47	12	1	0	0		Q3	53	12	0	0	0
Q4	47	13	0	0	0		Q4	53	11	1	0	0
Q5	46	13	1	0	0		Q5	51	12	2	0	0
Q6	46	13	1	0	0		Q6	54	10	1	0	0
Q7	47	12	1	0	0		Q7	53	11	1	0	0
Q8	48	11	1	0	0		Q8	54	9	2	0	0
Q9	48	10	2	0	0		Q9	54	10	1	0	0
Total number					60		Total number					65

*Note*: Details of Q1–Q9, please see Figure A5.

### Measurement Instruments Used in This Study

**Table A2:** Baseline equivalence of demographic subgroups.

Demographic Variables	N	Mean (SD) Scores of Pre-Training	*p*-Value
**By Age**			
<30	65	90.609 (9.480)	0.360
>=30	10	87.980 (6.529)	
**By Gender**			
Male	10	87.780 (12.061)	0.323
Female	65	90.640 (8.645)	
**By Ethnicity**			
Han	69	90.281 (9.001)	0.899
Other	6	90.000 (11.645)	
**By Highest Degree**			
Technical School or Below	56	90.662 (9.635)	0.747
Junior College	13	88.977 (8.242)	
Undergraduate	6	89.267 (6.952)	
**By Marital Status**			
Unmarried	66	90.630 (9.405)	0.309
Married	9	87.533 (6.761)	
**By Training Experience**			
0–3 Times	21	89.412 (8.917)	0.386
More Than 3 Times	54	90.754 (9.259)	
**By Training Length**			
Less Than 8 h	5	91.800 (6.573)	0.736
More Than 8 h	70	90.149 (9.339)	
